# Effect of PAMAM Dendrimers on Interactions and Transport of LiTFSI and NaTFSI in Propylene Carbonate-Based Electrolytes

**DOI:** 10.3390/polym12071595

**Published:** 2020-07-18

**Authors:** Rafał Konefał, Zuzana Morávková, Bartosz Paruzel, Vitalii Patsula, Sabina Abbrent, Kosma Szutkowski, Stefan Jurga

**Affiliations:** 1 Institute of Macromolecular Chemistry CAS, Heyrovského nám. 2, 162 06 Prague 6, Czech Republic; moravkova@imc.cas.cz (Z.M.); paruzel@imc.cas.cz (B.P.); patsula@imc.cas.cz (V.P.); abbrent@imc.cas.cz (S.A.); 2 NanoBioMedical Centre, Adam Mickiewicz University, Wszechnicy Piastowskiej 3, 61-614 Poznań, Poland; kosma.szutkowski@amu.edu.pl (K.S.); stjurga@amu.edu.pl (S.J.)

**Keywords:** PAMAM, dendrimers, LiTFSI, NaTFSI, propylene carbonate, conductivity, PFG NMR, diffusion coefficients, transport numbers, electrolyte

## Abstract

Poly(amidoamine) (PAMAM)-based electrolytes are prepared by dissolving the PAMAM half-generations G1.5 or G2.5 in propylene carbonate (PC), either with lithium bis(trifluoromethylsulfonyl)imide (LiTFSI) or sodium bis(trifluoromethylsulfonyl)imide (NaTFSI) salts. The solutions, designed for ion battery applications, are studied in terms of ions transport properties. Raman Spectroscopy reveals information about the interactions between cations and PAMAM dendrimers as well as full dissociation of the salts in all solutions. Pulsed-field gradient Nuclear Magnetic Resonance (PFG NMR), measured as a function of both temperature and PAMAM concentration, are obtained for the cation, anion, solvent, and dendrimer molecules using lithium (^7^Li), sodium (^23^Na), fluorine (^19^F), and hydrogen (^1^H) NMR, respectively. It was found that lithium diffusion is slow compared to the larger TFSI anion and decreases with PAMAM concentration due to interactions between cation and dendrimer. Comparison of conductivities calculated from diffusion coefficients using the Nernst–Einstein equation, with conductivity measurements obtained from Impedance Spectroscopy (IS), shows slightly higher IS conductivities, caused among others by PAMAM conductivity.

## 1. Introduction

Since their first appearance on the market in 1991 (Sony), lithium ion batteries have found their way into numerous applications in portable devices, ranging from smartphones, tablets, electronic cigarettes, torches, and cordless tools. More recently, due to environmental concerns, hybrid-electric and electric vehicles, have become more popular; therefore, the design and development of new high-performance high capacity ion batteries (IBs) becomes an urgent need [1,2,3]. An ion battery is a complex multicomponent device, where one of the critical components is an electrolyte [4]. It plays a crucial role in the ionic conductivity and, consequently, it affects the transport properties of the lithium Li^+^ ions (and, hence, high performance as well as long lifespan) in the battery. The electrolyte should not only uphold suitable ionic conductivity over a broad range of temperatures, but also possess good chemical stability and compatibility with electrode materials. The most popular and commonly used in commercial batteries are liquid electrolytes, based on the organic solvents (i.e., dimethyl carbonate, diethyl carbonate) with dissolved lithium salts (i.e., LiPF_6_) [1,5]. Unfortunately, those compositions are flammable and prone to rapid chemical decomposition reactions, which need to be controlled either by chemical additives, such as flame retardants, redox shuttles, and film-forming additives or electronic control devices (strict current and voltage control during charging and discharging cycles), that contribute to increased prices [6]. One of the alternatives to liquid electrolytes is the gel electrolyte, where lithium or sodium salts in the liquid electrolyte are mixed into a polymer material [1,7,8,9,10].

Dendrimers are a class of hyperbranched polymers with excellent monodispersity, globular shape, nanoscale size, interior pockets, well-defined molecular weight, and possible surface functionalities [11]. These properties endow them with great potential in miscellaneous applications, such as catalysis, supramolecular chemistry, host–guest interactions, drug or gene delivery, environmental science, and material science. A lot of studies have been conducted on the synthesis, characterization, and modification of novel dendrimers [12,13,14]. Among these, poly(amidoamine) (PAMAM) dendrimers have been the most studied, since their discovery by Tomalia in 1985 [15]. In general, the growth of PAMAM dendrimers is accomplished by alternating between two reactions, first, Michael addition of the amino-terminated surface onto methyl acrylate, resulting in an ester-terminated outer layer; second, coupling with ethylenediamine to achieve a new amino-terminated surface. Since they contain electronegative heteroatoms and surface functional groups that can be easily modified, PAMAM dendrimers are attractive for applications as electrolytes [16,17,18,19], single ionic conductors [20] as well as cathode binders [21] in IBs. Additionally, low glass transition temperature (≈−30 °C) and amorphous nature make them suitable materials for the modification of electrolytes [16,22]. 

In this contribution, the conductivity, interactions, and mobility (transport properties) of the ions in eighteen model electrolytes have been studied as a function of temperature, the generation of PAMAM dendrimer, and its concentration. To probe these properties, Impedance Spectroscopy has been used for ionic conductivity measurements, Raman Spectroscopy has been applied for study interactions between electrolytes components, and pulsed-field gradient Nuclear Magnetic Resonance (PFG NMR) Spectroscopy has been used for measurements of self-diffusion coefficients of individual species. These methods are commonly applied in these types of studies [18,23,24,25,26,27,28,29,30,31]. In all cases, propylene carbonate (PC) was used as the solvent due to its wide liquid range (−48.8–242 °C; MSDS) and good solvation properties. Two PAMAM dendrimer generations, G1.5 and G2.5, with *c* = 0, 10, 20, 40, and 60 wt% concentrations were dissolved in PC and finally, these solutions were used for electrolytes preparation with 1M lithium bis(trifluoromethylsulfonyl)imide (LiTFSI) or sodium bis(trifluoromethanesulfonyl)imide (NaTFSI) salts. A 1M salts concentration was chosen based on the fact that mobility and ionic conductivity typically show the highest value at this dissolution [32].

## 2. Materials and Methods 

### 2.1. Materials

Methyl acrylate (99%) and ethylenediamine (EDA) (99%) were purchased from Sigma-Aldrich (St. Louis, MO, USA); both chemicals were purified by distillation before use. Methanol (99%) and toluene (99%) were purchased from Lach-Ner (Neratovice, Czech Republic) and used as received. Propylene carbonate (extra pure) was purchased from ACROS Organics™ (Geel, Belgium); the bottle was opened under argon atmosphere and dried over 3 Å molecular sieves before use. LiTFSI 99%, Extra Dry and NaTFSI, min. 97% from abcr GmbH (Karlsruhe, Germany) were used as received. 

### 2.2. PAMAM Synthesis

The PANAM dendrimers were prepared according to the earlier published method [33]. 

#### 2.2.1. General Procedure for Preparation of Ester-Terminated PAMAM Dendrimers

The solution of amine-terminated dendrimer (or EDA in the very first step) in methanol was added dropwise under argon for 1 h to a stirred solution of methyl acrylate in methanol, previously cooled to 0–5 °C in an ice bath. The reaction mixture was further stirred at 0 °C for 1 h and then, at room temperature (RT) for 48 h. The finalization of the reaction was analyzed by ^1^H NMR. After that, the solvent and excess of reagents were removed under vacuum in the rotary-evaporator at 30 °C. The residue was additionally dried at 40 °C (13 Pa) for 5 h, resulting in colorless or yellow viscous syrup. Details of the performed reactions are shown in Table 1.

#### 2.2.2. General Procedure for Preparation of Amine-Terminated PAMAM Dendrimers

The solution of the ester-terminated dendrimer in methanol was added dropwise to the solution of EDA in methanol at 0 °C under vigorous stirring. The reaction mixture was stirred at RT for 96–132 h, and completion of the reaction was checked by ^1^H NMR. Then, the solvent was evacuated under vacuum in rotary-evaporator at 40 °C. The excess of EDA was removed by azeotropic distillation with toluene/methanol (9/1; *v/v*) mixture under vacuum (24 kPa) at 40 °C. The leftover toluene was removed by azeotropic distillation with methanol under reduced pressure at 40 °C and the obtained dendrimer was additionally dried in vacuum for 5 h (40 °C, 13 Pa). The resulting product was obtained as yellow syrup. Details of the performed reactions are shown in Table 2.

### 2.3. Ionic Conductivity Measurements

Ionic conductivity of investigated electrolytes was determined by impedance spectroscopy with the use of the Alpha-A High-Resolution Impedance Analyzer (Novocontrol Technologies, Montabaur, Germany) and conductivity cell. The cell consists of two parallel stainless-steel round electrodes with a polytetrafluoroethylene (PTFE) spacer, embodied in the polypropylene (PP) housing, and covered with a brass closing plate. The active part of the conductivity cell has a cylindrical shape with a diameter of 10 mm and a thickness of 5 mm. The cell with electrolyte was assembled and sealed in a glove box under an argon atmosphere and transferred to a cryostat of Novacontrol Quatro Cryosystem. The complex impedance measurements were conducted from 10 MHz to 0.01 Hz and at a small applied a.c. voltage (V_RMS_ = 1, 10, and 100 mV) in N_2_ atmosphere under isothermic conditions in a temperature range from 90 to 10 °C with a 10 °C step and cooling rate of 10 °C/15 min. The temperature of the sample was maintained by the flow of nitrogen gas through the heater in the Quatro Cryosystem. 

The conductivity is defined as the inverse of the sample resistivity and was calculated using Equation (1):

(1) $$\sigma =\frac{1}{\rho }=\frac{d}{AR_{s}}$$

where *σ* is the ionic conductivity, *ρ* is the resistivity of the solution, *d* is the thickness of the sample, *A* is the cross-sectional area of the active part of conductive cell, and *R_S_* is the bulk resistance of the measured solution. The bulk resistance of solutions was extracted from the Nyquist plot of acquired impedance data (as the real part of impedance when the imaginary part of impedance was zero) by graphical analyzes with the ZView software (Scribner Associates). This analysis was based on the determination of the intercept of (i) the low-frequency part of the high-frequency semicircle, and/or (ii) an inclined line in the low-frequency range (response of the ion diffusion or EDL formation), with the real axis [34]. 

### 2.4. Raman Spectroscopy

FT-Raman spectra of all samples were measured in sealed glass vessels (either NMR tubes or vials) with a Thermo Nicolet 6700 FTIR spectrometer with an FT Raman module NXR (Nd:YAG laser 1064 nm) in backscattering geometry, resolution 2 cm^−1^, and 1024 scans per spectrum. Relevant spectral regions were deconvoluted with Lorentzian lineshapes in Omnic 8.3.

### 2.5. Diffusion NMR Spectroscopy

All samples in PC solutions (1M LiTFSI or 1M NaTFSI and dendrimers concentrations *c* = 0, 10, 20, 40, and 60 wt%) were prepared inside the glove box under an argon atmosphere, filled, and capped into 5 mm Norell NMR Tubes. Next, to keep argon inside and prevent water sorption, the tubes were hermetically melt-sealed. For most of the samples, Lithium-7 (^7^Li), Sodium-23 (^23^Na), and Fluorine-19 (^19^F) pulsed field gradient echo diffusion experiments were performed on an Agilent 600 MHz NMR spectrometer using a 5 mm DOTY DSI-1372 probehead with a 25 T/m gradient coil, while proton (^1^H) measurements were done on a DOTY DSI-1374 probehead with a 25 T/m gradient coil. Four sample (1M NaTFSI with PAMAM G2.5) ^1^H and ^19^F diffusion experiments were performed using a Bruker Avance III 600 MHz NMR spectrometer using a DiffBB probehead and 40 A gradient amplifiers. In all cases, the gradient strength was varied in 16 steps. The data were collected every 10 °C from 90 to 10 °C. To correct any convection effects, a Pulsed Field Gradient Double-Stimulated Echo sequence was used. For achieving temperature stabilization, an additional 10 min waiting time was applied for all samples before the start of each experiment. For ^1^H, ^7^Li, and ^19^F, the pulse gradient time δ was 2 ms and the diffusion time Δ varied from 50 to 150 ms. From each experiment, the integrated intensities (*I*) as a function of the applied gradient (*g*) were obtained. Subsequently, diffusion coefficients (D) were then computed using single exponential decay by fitting of the Stejskal–Tanner equation [35]:

*I* = *I*_0_exp[−*Dg*^2^*γ*^2^*δ*^2^(Δ−*δ*/3)] (2)

The results obtained from the Agilent spectrometer were fitted with MNova 14 software, while experiments measured on a Bruker spectrometer were proceeded in TopSpin (Dynamic Center). 

Based on the diffusion coefficients, lithium and sodium cationic transport numbers were calculated:

(3) $$t_{+}=\frac{D_{+}}{D_{+}+D_{-}}$$

where *D_+_* and *D_−_* are the cation and anion diffusion coefficients, respectively. Moreover, the obtained diffusion coefficients allow calculation of ionic conductivity using the Nernst–Einstein equation:

(4) $$\delta_{NMR}=\frac{F^{2}\left[C\right]}{RT}\left(D_{+}+D_{-}\right)$$

where F is the Faraday constant (96485 C mol^−1^), *C* represents the concentration of the cation and anion (mol dm^−3^), R is the ideal gas constant (8.314472 J K^−1^ mol^−1^), and T is the temperature in K.

## 3. Results and Discussion

### 3.1. PAMAM Synthesis

**Figure 1:**
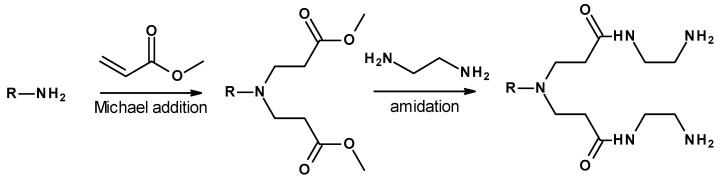
General scheme of PAMAM dendrimers preparation. The NH_2_-terminated structure represents full generations and the ester-terminated structure represents half-generations of PAMAM dendrimers.

In this work, methyl ester-terminated G1.5 and G2.5 PAMAM dendrimers were chosen for the electrolyte preparation. Selected dendrimers were prepared by a divergent synthesis approach, involving a reagent excess method starting from EDA by consecutive Michael addition and ester amidation reactions (Figure 1). The successful formation of each dendrimer generation was confirmed by ^1^H NMR and the obtained spectra were in agreement with the earlier published data [15,33]. The yield of dendrimers after synthesis was in the range of 95–98%. According to MALDI-TOF mass spectroscopy, the molecular weight of ester-terminated G1.5 and G2.5 PAMAM dendrimers was 2805 and 6007 Da, respectively. Obtained values correspond to theoretical ones, which were 2804 and 6004 Da for G1.5 and G2.5, respectively.

### 3.2. Conductivity

**Figure 2:**
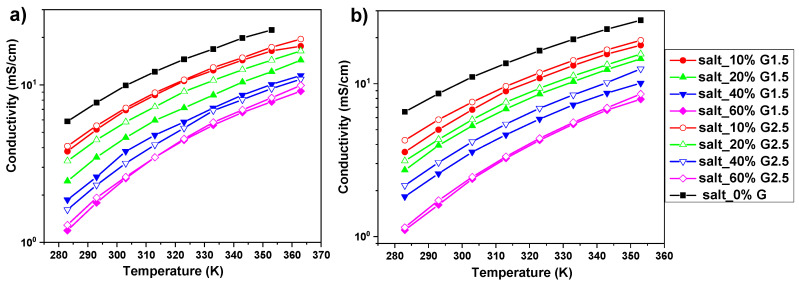
Temperature dependence of ionic conductivity for (**a**) LiTFSi salt and (**b**) NaTFSi salt-based electrolytes with 0, 10, 20, 40, and 60 wt% of PAMAM dendrimers with generations G1.5 and G2.5.

Ionic conductivities of the investigated electrolytes obtained from impedance spectroscopy are shown in Figure 2. In all cases, for both LiTFSI- and NaTFSI-based electrolytes, ionic conductivity decreased with the increasing concentration of PAMAM dendrimers (i.e., at 293 K in LiTFSI-based electrolytes, it decreases from *δ* = 7.7 mS/cm in a neat salt solution to *δ* = 1.8 mS/cm in the electrolyte with 60 wt% PAMAM G1.5 concentration) and increased with temperature (i.e., from *δ* = 3.8 mS/cm at 283K to 17.6 mS/cm at 363K in the case of the LiTFSI_10%G1.5 electrolyte). In general, slightly higher values of conductivities were recorded for electrolytes with the higher generation dendrimer G2.5. Additionally, some conductivity was observed in neat PAMAM dendrimer solutions (10 and 60 wt% in PC), which was at least one to two orders of magnitude lower than in salt solutions (i.e., at 293 K *δ* = 0.24 and 0.021 mS/cm for, respectively, 10 and 60 wt% concentrations of PAMAM G1.5 in PC—see more details in Figure S1 in the Supplementary Materials). This effect is in accordance with the literature data, where conductivity values for full generations of bulk PAMAM dendrimers were dependent on temperature and dendrimers size as observed by Mijović et al. [22]. In addition, the values of the conductivities of 1M LiTFSI and 1M NaTFSI solutions in PC are in good agreement with the literature. Nilsson et al., at 293K, observed the conductivity δ ≈ 4.5 and δ ≈ 1 mS/cm for 0.66 and 2.1M LiTFSI salt concentrations, and Geng et al., at 293K, detected the conductivity *δ* ≈ 7.5 mS/cm (in our case, *δ* = 8.6 mS/cm) [25,36]. 

### 3.3. Ion–Solvent–Dendrimer Interactions

**Figure 3:**
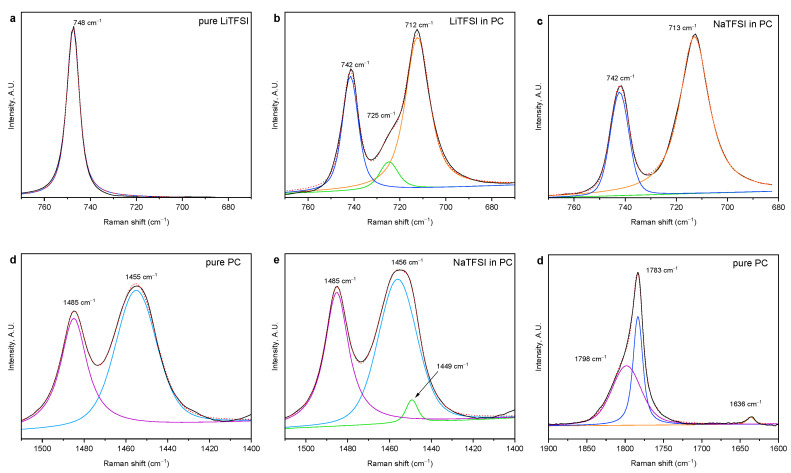

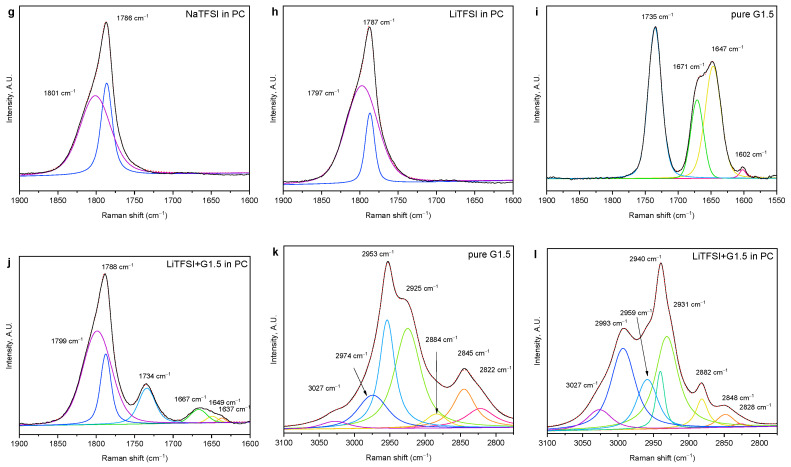
FT-Raman spectra of selected samples in selected spectral regions, including the results of the deconvolution to Lorentzian peaks: pure LiTFSI powder in the region 670–770 cm^−1^ (**a**), LiTFSI solution in PC in the region 670–770 cm^−1^ (**b**), NaTFSI solution in PC in the region 670–770 cm^−1^ (**c**), pure PC in the region 1400–1550 cm^−1^ (**d**), NaTFSI solution in PC in the region 1400–1550 cm^−1^ (**e**), pure PC in the region 1600–1900 cm^−1^ (**f**), NaTFSI solution in PC in the region 1600–1900 cm^−1^ (**g**), LiTFSI solution in PC in the region 1600–1900 cm^−1^ (**h**), pure G1.5 in the region 1550–1900 cm^−1^ (**i**), LiTFSI + G1.5 solution in PC in the region 1550–1900 cm^−1^ (**j**), pure G1.5 in the region 2750–3100 cm^−1^ (**k**), and LiTFSI + G1.5 solution in PC in the region 2750–3100 cm^−1^ (**l**). The experimental spectrum (black line) corresponds to the composition (red dotted line) of individual fitted peaks (other colors) labeled with their Raman shift. (Full FT-Raman spectra can be found in Supporting information Figure S2. All other zoom-ins with the deconvolution results can be found in Figures S3–S6).

In order to determine the interactions of the individual components, the chosen samples were analyzed using FT-Raman spectroscopy (Figure 3 and Figure S2). The CF_3_ deformation vibration of the TFSI anion is considered a marker of anion association, as it is located at 741 cm^−1^ in free dissociated salt, whereas on cation–anion interactions, it shifts to 748 cm^−1^ [37,38,39]. We have observed the peak located at 748 cm^−1^ only in solid salts; any band or shoulder at this location was not resolved in any of the salt-containing solutions (Figure 3a,b and Figure S3; the CF_3_ deformation vibrations are drawn with blue lines). Thereby, we can conclude that the salts are fully dissociated. No further changes to this TFSI anion band were observed on the addition of PAMAM dendrimers.

The deformation vibration of the PC ring at 712 cm^−1^ is very sensitive to interactions with lithium. Lithium ion coordinates to the carbonyl and both ether oxygen atoms, “sitting” above the PC ring, which leads to stiffening of the PC ring and a shift of this ring vibration to a higher frequency [40,41,42]. We have observed the formation of such coordination band at 725 cm^−1^ upon the addition of lithium (Figure 3b and Figure S3). According to Allen et al., the coordination number of lithium to PC can be calculated from the intensities of the two bands as C=1.43I725I712 [40]. This gives us the coordination number for LiTFSI solution in PC equal to 16.6. Upon addition of G1.5, it drops to 1.8 and upon addition of G2.5 to 2.7, indicating that lithium ions interact with PAMAM more strongly than with PC.

This PC ring vibration shift was not observed upon addition of sodium salt (Figure 3c), signifying that such interaction is not operative for sodium. However, in the case of sodium, we have observed minor changes in CH_3_ deformation band (Figure S4) and carbonyl stretching band (Figure S5). Pure PC has two peaks in the C–H region between 1400 and 1500 cm^−1^ (Figure 3d): an anti-symmetrical CH_3_ deformation peak around 1455 cm^−1^ and O–CH_2_ deformation peak around 1485 cm^−1^ [17,43]. On addition of both lithium and sodium salts, a frequency shoulder forms at 1449 cm^−1^ (Figure 3e and Figure S4). This shoulder is present even after the addition of PAMAM (Figure S4), indicating that PC–cation interaction remains to some extent. In the carbonyl stretching region, PC has three bands (Figure 3f and Figure S5): its carbonyl stretching region is split to symmetrical (1783 cm^−1^) and anti-symmetrical (1798 cm^−1^) vibration peaks [44] and a small band at 1636 cm^−1^. The position of the symmetrical part at 1783 cm^−1^ is sensitive to molecular order [44]. This band shifts upon addition of salts to 1786 cm^−1^ (NaTFSI, Figure 3g) and 1787 cm^−1^ (LiTFSI, Figure 3h) and to 1788 cm^−1^ in full electrolytes (Figure S5), further indicating a solvent–cation interaction. The ratio of the two bands also changes—more strongly upon addition of LiTFSI than NaTFSI (Table 3). Changes in the C–H stretching region are also observed (Figure S6): all bands in this region shift slightly. These observations, which are in agreement with previously published calculations [45,46,47], indicate that both ions interact weakly with the carbonyl oxygen of PC, forming a solvation shell with the carbonyls directed to the ion in the center and methyl groups on the surface. This changes the local polarity around the methyl groups, leading to the shift of the anti-symmetrical CH_3_ deformation vibration and C-H stretching vibrations. 

The carbonyl stretching region is also relevant for studying the interactions of PAMAM. PAMAM has two types of carbonyl groups: ester end-groups with carbonyl stretching frequency of 1735 cm^−1^ and amide inner groups with carbonyl stretching band split into two maxima at 1647/1645 and 1671/1670 cm^−1^ for G1.5/G2.5, respectively (Figure 3i and Figure S5) [17,48]. The band at lower Raman shift, that slightly dominates in the pure dendrimer (Figure 3i), belongs to the hydrogen-bonded groups of concentrated amides [43], whereas upon dilution or disturbance of the dendrimer structure, the hydrogen bonds are broken, leading to an increase in the band of free amide (Figure 3j). Hydrogen bond breakage takes place already upon dilution of PAMAM with PC, but upon addition of salts, it goes even further (Figure S5). This effect is stronger for NaTFSI than LiTFSI as demonstrated in Table 3. 

When the area of the ester band (compared to the amide bands) is observed, it is seen to increase upon dilution and addition of salts, the effect being strongest for LiTFSI. An interesting fact is that the ratio of the ester to amide band areas in the corresponding G1.5 and G2.5 samples remains around 1.35, with an exception of the addition of NaTFSI, where this ratio changes to 1.65. We speculate that G2.5 allows the incorporation of larger species within its structure, so G1.5 is more disturbed by the incorporation of sodium cation. The symmetrical carbonyl stretching band of PC shifts upon the addition of PAMAM dendrimers to 1785 cm^−1^ (Figure 3j and Figure S5, Table 3), proving the solvation interaction between PAMAM and PC. The ratio of the two PC carbonyl bands also changes more strongly upon the addition of G1.5 than G2.5 (Table 3). The small peak at 1601 cm^−1^ in the spectra of pure dendrimers belongs to the N–H deformation vibration representing intra-molecular hydrogen bonding [43]. However, it remains unresolved in the solutions with PC. On the other hand, a peak around 1635 cm^−1^ is well resolved and stronger than in pure PC, which is probably caused by the contribution from the deformation of the N–H free of H-bonding. 

The C–H stretching vibrations of PAMAM (Figure 3k,l and Figure S6) are also influenced by dilution and addition of salts, however, only the symmetrical stretching vibrations of the CH_2_ groups at 2822 and 2845 cm^−1^ [49] can be reliably analyzed as they do not overlap with the C–H stretching bands of PC. Both bands shift to higher frequencies upon mixing. Furthermore, the ratio of the two bands decreases with mixing. LiTFSI has a stronger effect, while NaTFSI and PC exhibit approximately the same influence (Table 4). This ratio is slightly higher for G2.5 than G1.5 in pure PAMAM but reverses with the addition of salt. We can speculate that this is again connected to the ability of the G2.5 molecule to incorporate larger structures. 

These observations indicate that the cations enter the dendrimer structure, although with a different kind of interaction. Sodium influences the amide carbonyl stretching of PAMAM more, whereas lithium disturbs the CH_2_ group’s stretching. We can speculate that PAMAM branches bend more closely around lithium than sodium. 

### 3.4. NMR Diffusometry

Pulsed Field Gradient Double-Stimulated Echo NMR diffusion measurements [35,50] were used to characterize the individual motion of cation, anion, solvent, and dendrimer. The obtained self-diffusion coefficients allow us to determine important electrolyte properties such as conductivity and transference numbers. The four resonant frequencies (^1^H, ^7^Li, ^23^Na, and ^19^F) as a function of temperature were used in order to follow the diffusion of PC, PAMAM, cations, and anion, respectively. For all prepared electrolytes and temperatures, ^1^H, ^7^Li, and ^19^F self-diffusion coefficients were measured without complications. In the case of ^23^Na cation, the diffusion coefficients were obtained only for the “NaTFSI_0%G” electrolyte (1M solution of NaTSI in PC) in a 40–90 °C temperature range. The inability to measure ^23^Na diffusion coefficients in electrolytes with PAMAM dendrimers is caused by a large quadrupolar moment of this nuclei, as well as very fast spin–spin relaxation times (≤3 ms) and weak signal to noise ratio, caused by higher viscosity of PAMAM-based electrolytes. A similar effect was observed by D. Morales et al. in NaPF_6_ -tetraglyme electrolytes [23].

**Figure 4:**
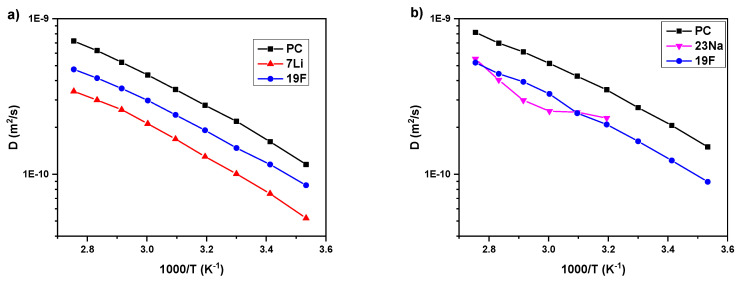
Temperature dependence of NMR self-diffusion coefficients for the LiTFSI_0%G (**a**) and NaTFSI_0%G (**b**) electrolytes (without PAMAM). The solid lines are guides for the eye.

The diffusion coefficients for ^1^H (PC), ^7^Li (lithium cation), ^23^Na (sodium cation), and ^19^F (TFSI anion) nuclei in LiTFSI_0%G and NaTFSI_0%G electrolytes as a function of temperature are shown in Figure 4. In both samples, the PC molecules have higher mobility than either the cation or anion. Comparing the solutions, there is a visible difference in cation behavior. In the case of lithium-based electrolyte, cations have the slowest mobility (*D*_Li_ = 1.30e^−10^ m^2^/s, *D*_TFSI_ = 1.91e^−10^ m^2^/s, and *D*_PC_ = 2.78e^−10^ m^2^/s at 313 K) despite being the smallest species, while larger sodium cations have higher diffusion coefficients similar to TFSI anions (*D*_Na_ = 2.30e^−10^ m^2^/s, *D*_TFSI_ = 2.09e^−10^ m^2^/s, and *D*_PC_ = 3.49e^−10^ m^2^/s at 313K). This is the result of different interactions between the cations and PC molecules. As was observed by Raman Spectroscopy, the smaller Li cations interact strongly with carbonyl groups of PC and the effective radius of lithium ions becomes large due to the resulting solvation shell. This effect of diffusion order (PC/anion/cation), revealed by PFG NMR was also observed in the literature for similar systems (LiTFSI/PC), as well as for other solvents or lithium salts (LiBF_4_/PC; PYR_14_TFSI/PC/LiTFSI; LiTFSI/GBL; LiBETI/GBL) [25,28,51,52,53,54]. As was mentioned above, in NaTFSI_0%G electrolyte (Figure 4b), the solvent has higher mobility concerning the ions and the most visible difference is that all components are more mobile than in the Li-based electrolyte. Particularly, sodium cation has higher diffusion coefficients than Li. This behavior is an effect of weaker interactions between Na and PC, as observed by Raman spectroscopy. Additionally, similar values of diffusion coefficients of Na and TFSI ions (*D*_Na_ ≈ 3.00e^−10^ m^2^/s) were observed in NaTFSI-glyme electrolytes by Carbone et al. [29].

**Figure 5:**
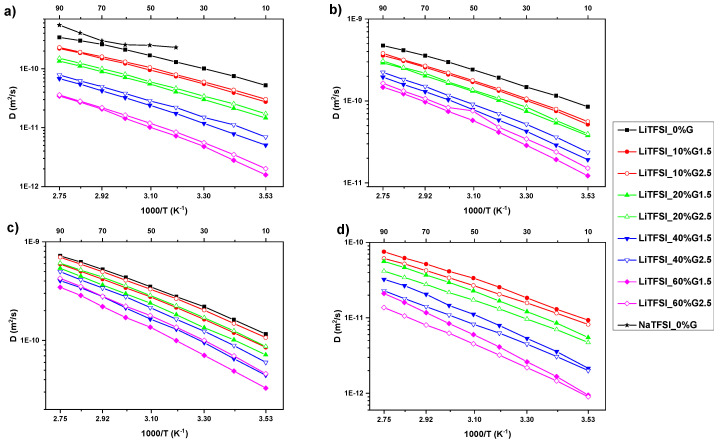
Temperature dependence of NMR self-diffusion coefficients for the LiTFSI-based electrolytes for Li cation (**a**), TFSI anion (**b**), PC molecules (**c**), and PAMAM molecules (**d**). The solid lines are guides for the eye.

The effect of the two PAMAM generations (G1.5 and G2.5) and different concentrations on temperature behavior of all components (Li cation, TFSI anion, PC, and PAMAM) in LiTFSI-based electrolytes determined by PFG NMR is shown in Figure 5. Results obtained for individual samples are shown in the Supplementary Materials (Figures S7–S22). Several trends arise from these data. Firstly, the values of all diffusion coefficients decrease with the increasing PAMAM concentration (i.e., at 293K for: Li^+^ from *D*_Li_ = 7.50e^−11^ m^2^/s in electrolyte without PAMAM to *D*_Li_ = 2.80e^−12^ m^2^/s in electrolyte with 60 wt% of PAMAM G1.5; TFSI anion from *D*_TFSI_ = 1.16e^−10^ m^2^/s in electrolyte without PAMAM to *D*_TFSI_ = 1.92e^−11^ m^2^/s in electrolyte with 60 wt% of PAMAM G1.5; PC molecules from *D*_PC_ = 1.62e^−10^ m^2^/s in electrolyte without PAMAM to *D*_PC_ = 4.89e^−11^ m^2^/s in electrolyte with 60 wt% of PAMAM G1.5; PAMAM molecules from *D*_PAMAM G1.5_ = 1.30e ^−11^ m^2^/s in electrolyte with 10 wt% of PAMAM G1.5 to *D*_PAMAM G1.5_ = 1.67e^−12^ m^2^/s in electrolyte with 60 wt% of PAMAM G1.5; finally, from *D*_PAMAM G2.5_ = 1.15e^−11^ m^2^/s in electrolyte with 10 wt% of PAMAM G2.5 to *D*_PAMAM G2.5_ = 1.46e^−12^ m^2^/s in electrolyte with 60 wt% of PAMAM G2.5). This is reasonable as increasing viscosity usually results in lower mobility of the system [23,25,29]. Secondly, unexpected slightly higher diffusion coefficients of cation, anion, and solvent are detected for electrolytes with the higher PAMAM generation. This can be explained either by slightly weaker interactions between PAMAM G2.5 dendrimer and cation, anion, and PC, or lower penetration of salt and solvent into PAMAM G2.5 than PAMAM G1.5 structure. Moreover, it may be also caused by higher mobility of the penetrated ion in PAMAM as G2.5 has larger pockets inside its structure. The longer branches of G2.5 would then be more mobile within the pockets. Thirdly, the strongest decrease in diffusion coefficient values with increasing PAMAM concentration is observed for Li cation, in comparison to TFSI anion and PC (see listed values above). This is the result of penetration by Li cation into the PAMAM structure observed by Raman spectroscopy. Fourthly, as expected due to the dendrimer size, we observe lower diffusion coefficients for PAMAM G2.5 than G1.5. The same behavior was observed for anion, PC, and PAMAM in NaTFSI-based electrolytes, as presented in the Supplementary Materials (Figures S23–S25). Based on these results, it can be concluded that Li cations use the ion-hopping mechanism to move through the electrolyte [1].

**Figure 6:**
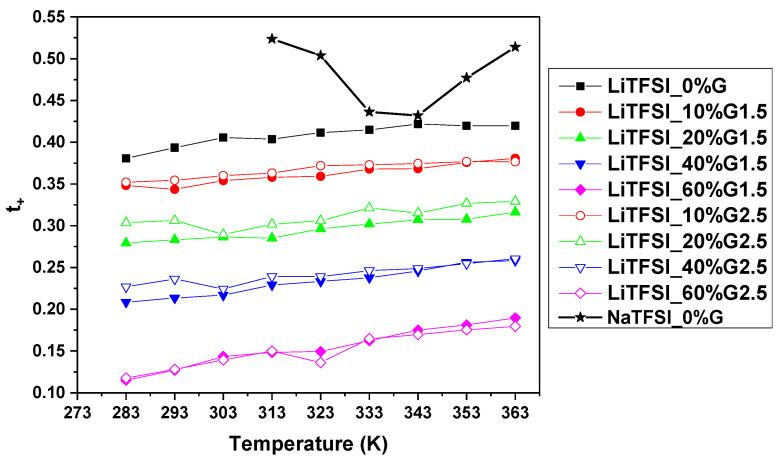
Temperature dependence of cation transference numbers for NaTFSI_0%G electrolyte and LiTFSI electrolytes with different amounts of PAMAM. The solid lines are guides for the eye.

Transport properties of the electrolytes can also be determined by transference (transport) numbers [25,29]. Transport numbers *t*_+_, derived from diffusion coefficients using Equation (3) for all LiTFSI-based and NaTFSI_0%G electrolytes, are presented in Figure 6. From the figure, it follows that values of Na^+^ transport numbers (*t*_+Na_ ≈ 0.5) are higher than for Li^+^ (*t*_+Li_ ≈ 0.4), when comparing NaTFSI_0%G and LiTFSI_0%G electrolytes. The Na^+^ values about 0.5 correlate well with the literature ones (0.5) observed in NaTFSI-glyme electrolytes by Carbone et al. [29], and thus, the electrolyte can be considered suitable for application in sodium batteries. The values of Li^+^ transport numbers in LiTFSI_0%G electrolyte are also practically the same (*t*_+Li_ ≈ 0.39), as obtained by Sethurajan et al. [55] using the same method. Similarly to the Li self-diffusion coefficients (Figure 5), the addition of PAMAM dendrimers results in a decrease in Li^+^ transport number values (i.e., at 293K from *t*_+Li_ = 0.39 in electrolyte without PAMAM to *t*_+Li_ = 0.13 in the electrolyte with 60 wt% of PAMAM G1.5); the higher the PAMAM amount, the lower the *t_Li_*_+_. This effect shows that Li cations strongly interact with PAMAM dendrimers and confirms results obtained by Raman Spectroscopy.

**Figure 7:**
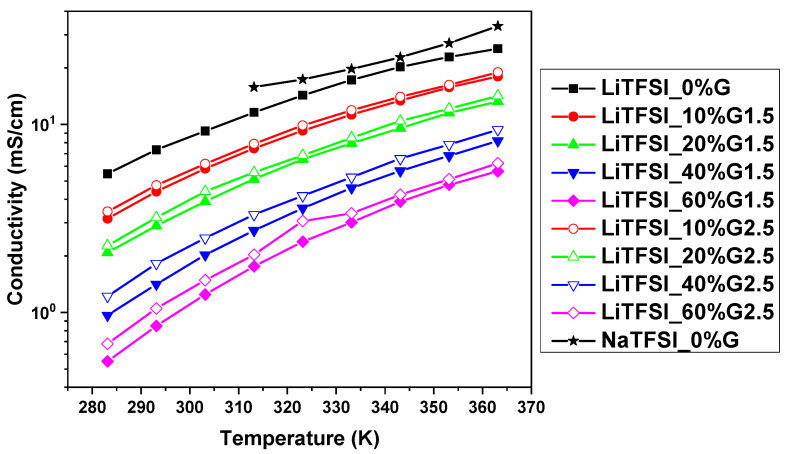
Temperature dependence of ion conductivities calculated from PFG NMR self-diffusion coefficients for NaTFSI_0%G electrolyte and LiTFSI electrolytes with different amounts of PAMAM. The solid lines are guides for the eye.

Conductivity given by assumption of complete ion dissociation is calculated using the Nernst–Einstein equation (4) and the results are presented in Figure 7. As the diffusion increases with temperature, so does the conductivity (i.e., from *δ_D_* = 3.16 mS/cm at 283K to 17.99 mS/cm at 363K in the case of the LiTFSI_10%G1.5 electrolyte), as is also observed by Impedance Spectroscopy. Moreover, similarly to conductivity measured by IS, Li diffusion coefficients and Li^+^ transport numbers, the addition of PAMAM dendrimers results in a decrease in the calculated conductivity values (i.e., at 293K in LiTFSI-based electrolytes, they decrease from *δ_D_* = 7.34 mS/cm in a neat salt solution to *δ_D_* = 0.85 mS/cm in the electrolyte with 60 wt% PAMAM G1.5 concentration). These results are also in agreement with the fact that in electrolytes with higher dendrimer generation, conductivity values are slightly higher. Comparing these values to IS conductivity measurements (Figure 2a), a striking difference is revealed: the values calculated using the Nernst–Einstein equation for solutions with various concentrations of dendrimers are lower than those obtained for respective solutions recorded by IS (i.e., *δ_D_* = 4.40 and *δ* =5.21 mS/cm at 393K in the case of the LiTFSI_10%G1.5 electrolyte). On the one hand, this is in contrast to results obtained for liquid electrolytes presented in the literature [23,25,28,29], but on the other hand, it is understandable, taking into account the recorded ionic conductivities of the neat PAMAM solution (see Figure S1). This influences the impedance measurement (in IS, total conductivity of the electrolyte is measured) and increases ionic conductivities of electrolytes in contrast to conductivities calculated from diffusion coefficients, where only the ions are taken into account. Finally, the average diffusion coefficients of Li cations from NMR studies are influenced by their interactions with dissolved dendrimers, as was observed by Raman Spectroscopy. This can lead to slightly underestimated values of the diffusion coefficients of non-interacting ions, which contribute the most to the measured conductivity. The average diffusion coefficients additionally decrease the values of conductivity calculated by the Equation (3) in comparison to the values obtained from the impedance measurement.

## 4. Conclusions

We used PFG NMR diffusion, Raman Spectroscopy, and Impedance Spectroscopy measurements for detailed characterization of interactions and transport properties as a function of temperature, PAMAM dendrimer concentration and generation in LiTFSI and NaTFSI–PC based solutions for application as battery electrolytes.

Raman Spectroscopy shows complete dissociation of LiTFSI and NaTFSI salts in the solutions, as well as gives information about strong interactions between Li^+^ and Na^+^ cations and PAMAM dendrimers. The comparison of self-diffusion coefficients obtained for Li^+^ and Na^+^ cations in electrolytes without PAMAM shows lower values for lithium ion, which is caused by stronger interactions of Li^+^ than Na^+^ with PC and results in higher solvation shell of lithium cation. Furthermore, lithium ions are found to be slower than PC and fluorine species, due to strong interactions between cations and PAMAM molecules. Additionally, the values of Li^+^ self-diffusion coefficients are higher than obtained for PAMAM dendrimers, which suggests the existence of the hopping transport mechanism of Li^+^ between PAMAM molecules. Moreover, the values of self-diffusion coefficients for all electrolyte components decrease with an increasing PAMAM concentration, which is a result of the increased viscosity of the solutions. The Li^+^ cationic transport numbers calculated from the self-diffusion coefficients decrease with the increasing PAMAM concentrations. This has been attributed to an increasing number of positions (places) for interactions with the cations. The Nernst–Einstein equation is used to calculate conductivity from NMR diffusion coefficient measurements. Assumably, the calculated conductivity is smaller than the measured values because NMR conductivity shows only ion mobility, while IS measures the overall conductivity of the whole electrolyte. The higher values may be attributed to the dendrimer—neat PAMAM solutions have been shown to possess intrinsic conductivity.

## Figures and Tables

**Table 1:** Synthesis of ester-terminated PAMAM dendrimers.

Generation of Obtained Dendrimer	Composition of the Reaction Mixture					Reaction time, h
	Starting Dendrimer Solution			Methyl Acrylate Solution		
	Generation	mmol	Methanol, mL	Methyl Acrylate, mmol	Methanol, mL	
G- 0.5	EDA	40	20	225	20	48
G0.5	G0	20	20	200	20	48
G1.5	G1	20	40	400	50	48
G2.5	G2	16	120	650	100	48

**Table 2:** Synthesis of amine-terminated PAMAM dendrimers.

Generation of Obtained Dendrimer	Composition of the Reaction Mixture					Reaction Time, h
	Starting Dendrimer Solution			EDA Solution		
	Generation	mmol	Methanol, mL	EDA, mmol	Methanol, mL	
G0	G- 0.5	40	30	2000	160	96
G1	G0.5	20	100	1200	120	96
G2	G1.5	16	120	6800	400	132

**Table 3:** Positions and areas normalized to the summary area of bands connected with the component, to which the vibration belongs. Carbonyl stretching region.

	PC		Amide H-Bonded		Amide Free		Ester		Amide/Ester	PC Sym.		PC Anti-Sym.	
	pos.	area	pos.	area	pos.	area	pos.	area		pos.	area	pos.	area
PC	1636	2								1783	40	1798	58
NaTFSI+PC	1636	0								1786	32	1801	68
LiTFSI+PC	1636	0								1787	18	1797	82
G1.5			1647	37	1671	19	1735	43	76				
G2.5			1645	44	1670	18	1735	35	56				
G1.5 in PC	1636	4.5	1647	12	1666	39	1735	49	96	1785	29	1798	67
G2.5 in PC	1636	4.1	1648	19	1666	39	1735	41	71	1785	34	1798	62
NaTFSI+G1.5+PC	1638	0.3	1649	9	1667	32	1734	59	142	1788	26	1801	73
NaTFSI+G2.5+PC	1636	0.5	1648	11	1666	42	1735	46	86	1788	25	1800	74
LiTFSI+G1.5+PC	1637	0.9	1649	7	1667	23	1734	70	231	1788	24	1799	75
LiTFSI+G2.5+PC	1636	0.9	1649	10	1666	25	1735	64	180	1787	22	1798	77

**Table 4:** Positions and area ratios of symmetrical CH_2_ stretching bands of PAMAM.

	Position	Position	Area Ratio
G1.5	2822	2845	66
G2.5	2822	2845	75
G1.5 in PC	2826	2846	46
G2.5 in PC	2827	2846	46
NaTFSIinPC+G1.5	2826	2846	43
NaTFSIinPC+G2.5	2827	2847	41
LiTFSIinPC+G1.5	2828	2848	22
LiTFSIinPC+G2.5	2828	2848	20

## Supplementary Materials

The following are available online at https://www.mdpi.com/2073-4360/12/7/1595/s1, Figure S1: Ionic conductivity for propylene carbonate solutions of PAMAM dendrimers G1.5 and G2.5 as a function of temperature. Figure S2: FT-Raman spectra of the electrolytes formed by dendrimers G1.5 and G2.5 in PC with LiTFSI or NaTFSI, pure samples as well as their solutions in PC are provided for comparison. Figures S3–S6: Deconvolutions of the FT-Raman spectra. Figures S7–S22: NMR self-diffusion coefficients for the individual electrolytes. Figures S23–S25: Comparisons of the individual species NMR self-diffusion coefficients for the NaTFSI-based electrolytes.
