# Supplementary material for: Effect of PAMAM Dendrimers on Interactions and Transport of LiTFSI and NaTFSI in Propylene Carbonate-Based Electrolytes
Source: Polymers (Basel). 2020 Jul 18;12(7):1595. doi: 10.3390/polym12071595 (PMC7407142; doi:10.3390/polym12071595)
Supplement: Supplementary file 1 [file polymers-12-01595-s001.pdf]

Supplementary Material

Effect of PAMAM dendrimers on interactions and transport of LiTFSI and NaTFSI in propylene carbonate-based electrolytes

Rafał Konefał [1,*], Zuzana Morávková [1], Bartosz Paruzel [1], Vitalii Patsula [1], Sabina Abbrent[1], Kosma Szutkowski[2] and Stefan Jurga [2]

[1] Institute of Macromolecular Chemistry CAS, Heyrovského nám. 2, 162 06 Prague 6, Czech Republic;
[2] NanoBioMedical Centre, Adam Mickiewicz University, Wszechnicy Piastowskiej 3, 61-614 Poznań, Poland;
* Correspondence: konefal@imc.cas.cz (R.K.)

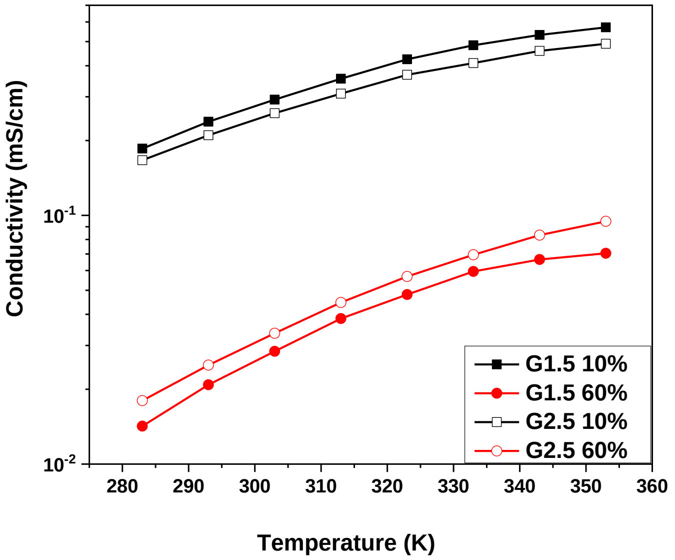

Figure S1. Ionic conductivity for propylene carbonate solutions of PAMAM dendrimers G1.5 and G2.5 as a function of temperature.

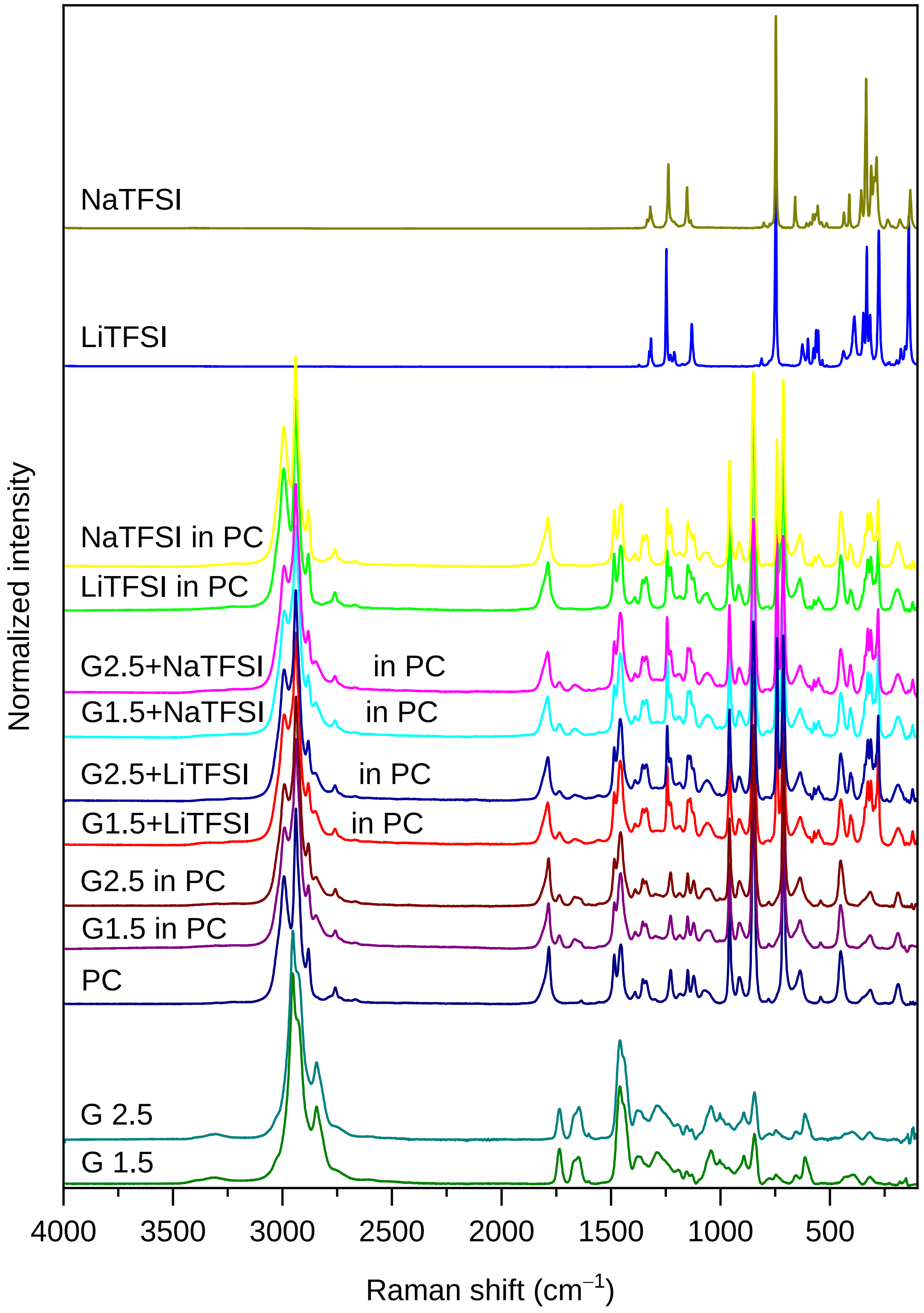

Figure S2. FT-Raman spectra of the electrolytes formed by dendrimers G1.5 and G2.5 in PC with LiTFSI or NaTFSI, pure samples as well as their solutions in PC are provided for comparison. The spectra are baseline corrected, normalized and shifted for clarity.

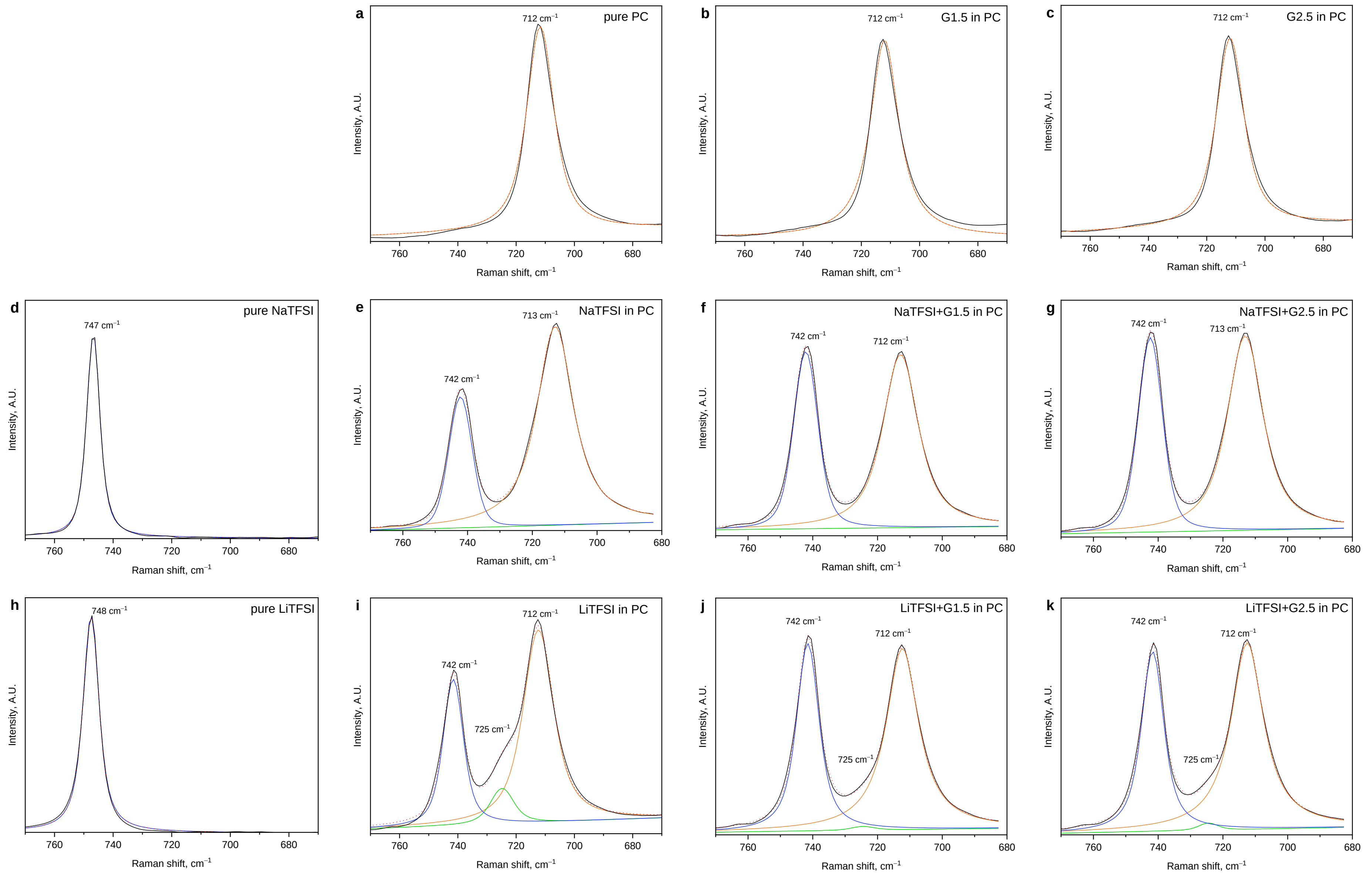

Figure S3. Deconvolutions of the spectral region 670—770 cm^{-1} of the FT-Raman spectra of pure PC (a), G1.5 in PC (b), G2.5 in PC (c), pure NaTFSI (d), NaTFSI in PC (e), NaTFSI with G1.5 in PC (f), NaTFSI with G2.5 in PC (g), pure LiTFSI (h), LiTFSI in PC (i), LiTFSI with G1.5 in PC (j) and LiTFSI with G2.5 in PC (k). Experimental spectrum (black line) corresponds to the composition (red dotted line) of individual fitted peaks (other colors).

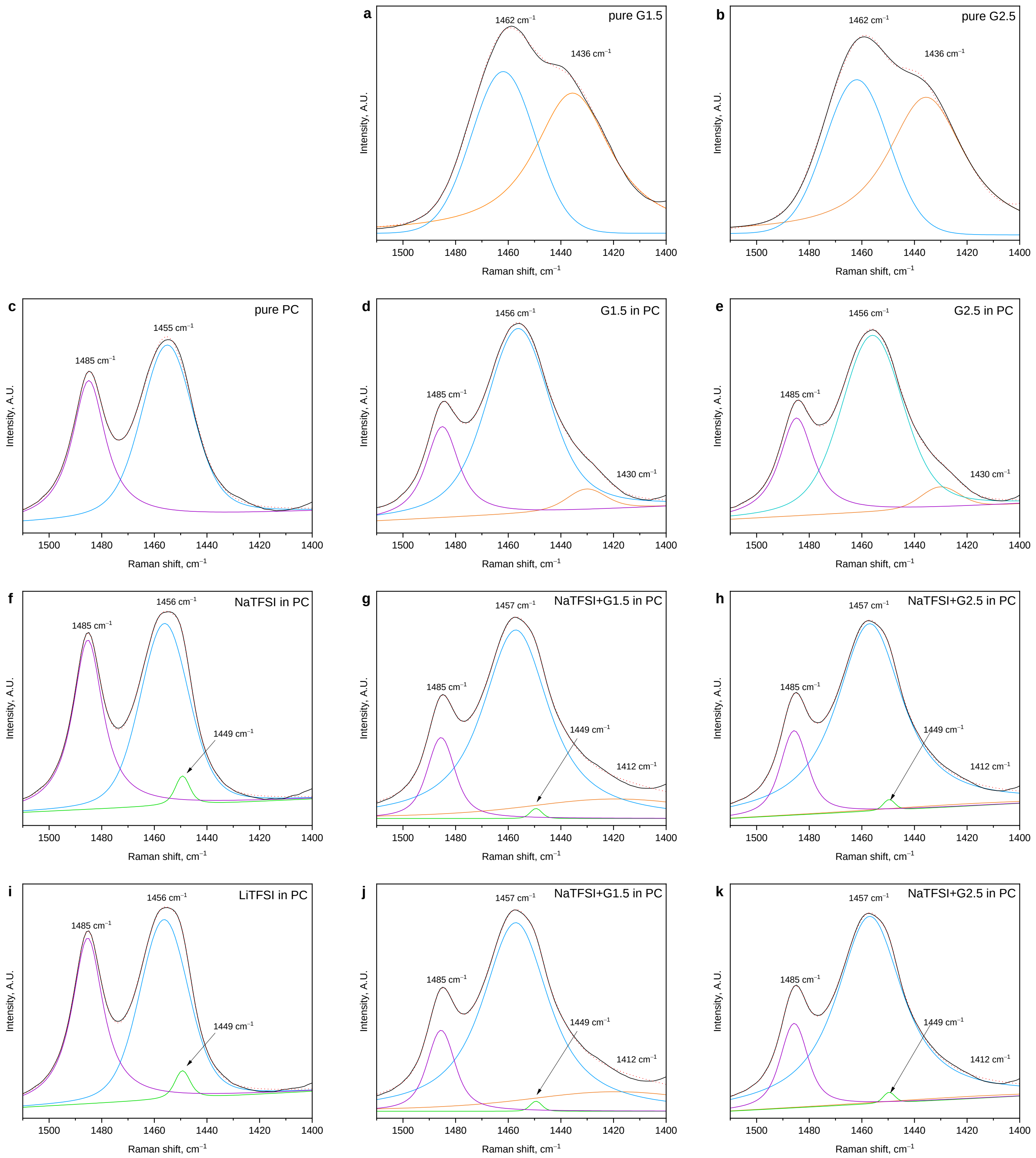

Figure S4. Deconvolutions of the C—H deformation region of the FT-Raman spectra of pure G1.5 (a), pure G2.5 (b), pure PC (c), G1.5 in PC (d), G2.5 in PC (e), NaTFSI in PC (f), NaTFSI with G1.5 in PC (g), NaTFSI with G2.5 in PC (h), LiTFSI in PC (i), LiTFSI with G1.5 in PC (j) and LiTFSI with G2.5 in PC (k). Experimental spectrum (black line) corresponds to the composition (red dotted line) of individual fitted peaks (other colors).

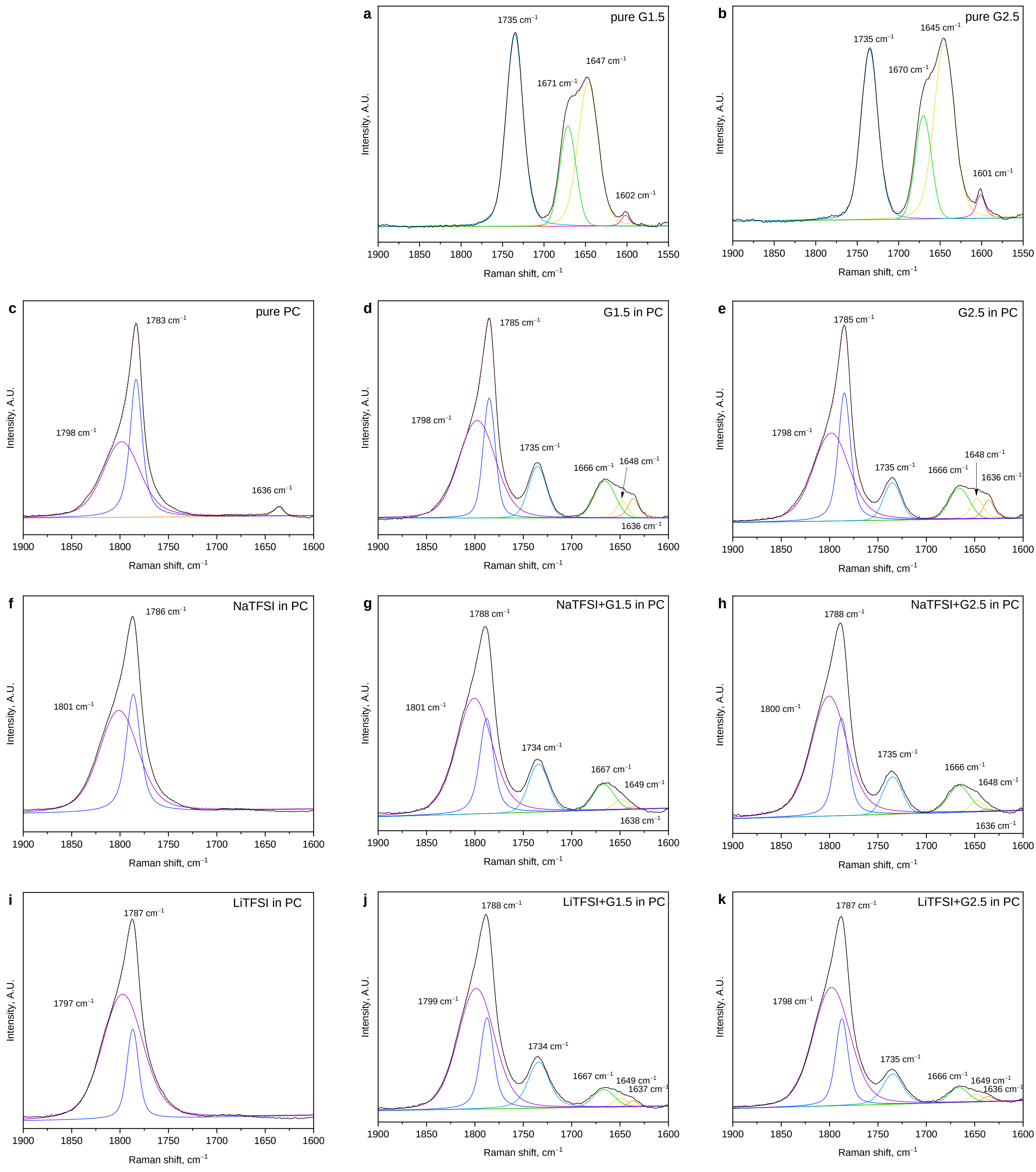

Figure S5. Deconvolutions of the carbonyl stretching region of the FT-Raman spectra of pure G1.5 (a), pure G2.5 (b), pure PC (c), G1.5 in PC (d), G2.5 in PC (e), NaTFSI in PC (f), NaTFSI with G1.5 in PC (g), NaTFSI with G2.5 in PC (h), LiTFSI in PC (i), LiTFSI with G1.5 in PC

(j) and LiTFSI with G2.5 in PC (k). Experimental spectrum (black line) corresponds to the composition (red dotted line) of individual fitted peaks (other colors).

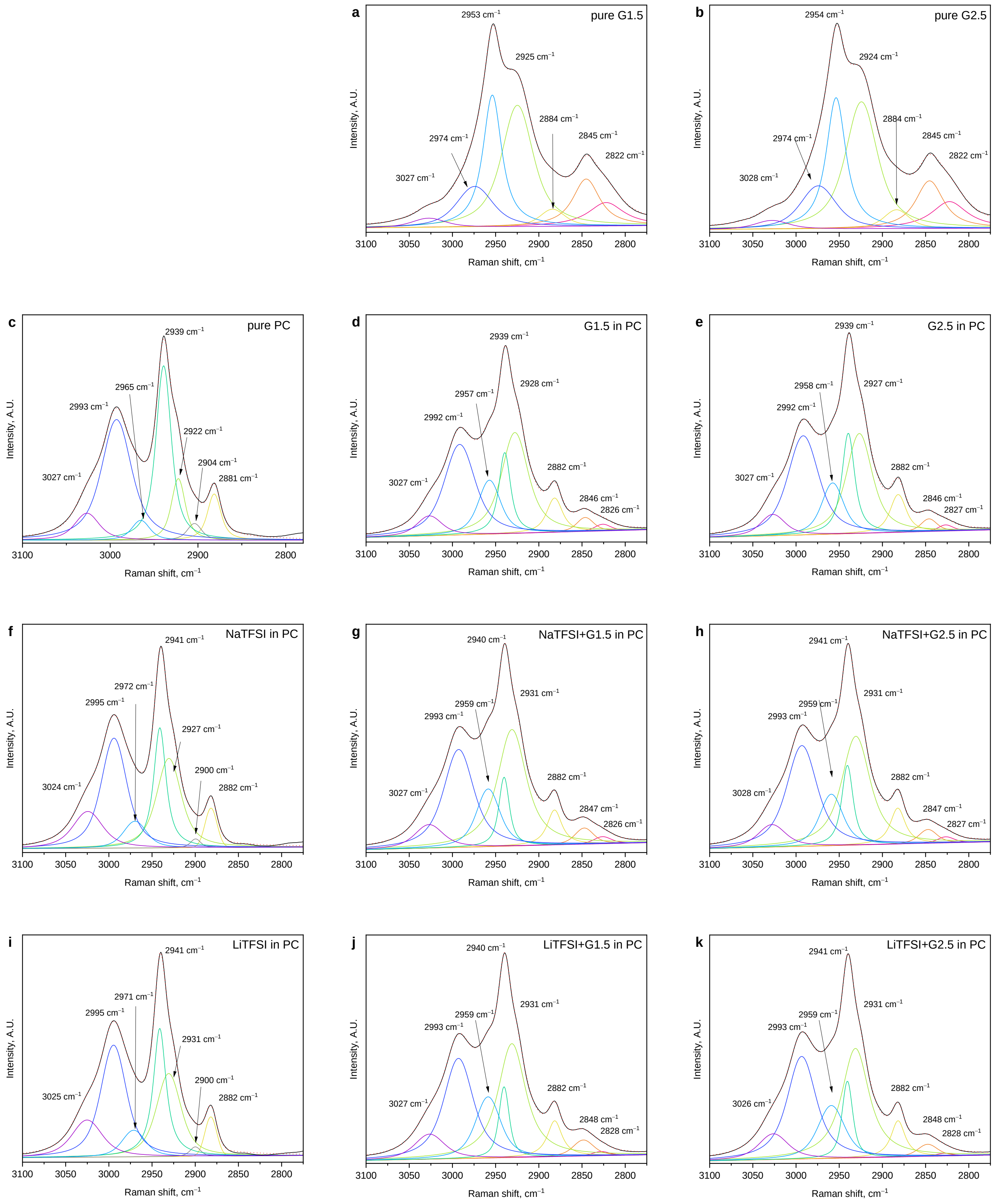

Figure S6. Deconvolutions of the C—H stretching region of the FT-Raman spectra of pure G1.5 (a), pure G2.5 (b), pure PC (c), G1.5 in PC (d), G2.5 in PC (e), NaTFSI in PC (f), NaTFSI with G1.5 in PC (g), NaTFSI with G2.5 in PC (h), LiTFSI in PC (i), LiTFSI with G1.5 in PC (j) and LiTFSI with G2.5 in PC (k). Experimental spectrum (black line) corresponds to the composition (red dotted line) of individual fitted peaks (other colors).

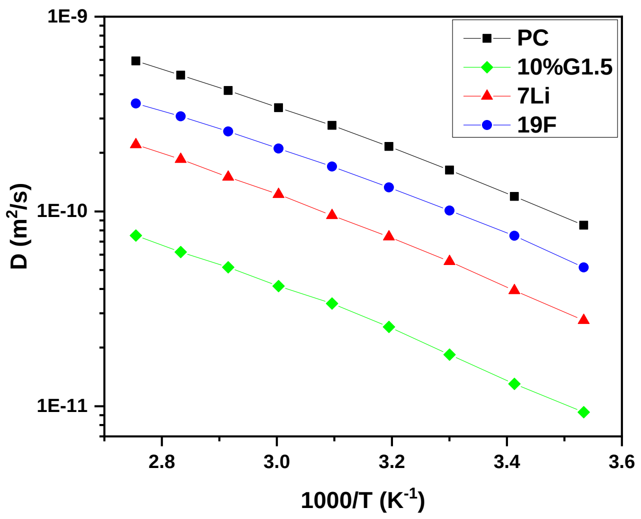

Figure S7. Temperature dependence of NMR self-diffusion coefficients for the LiTFSI_10%G1.5 electrolyte.

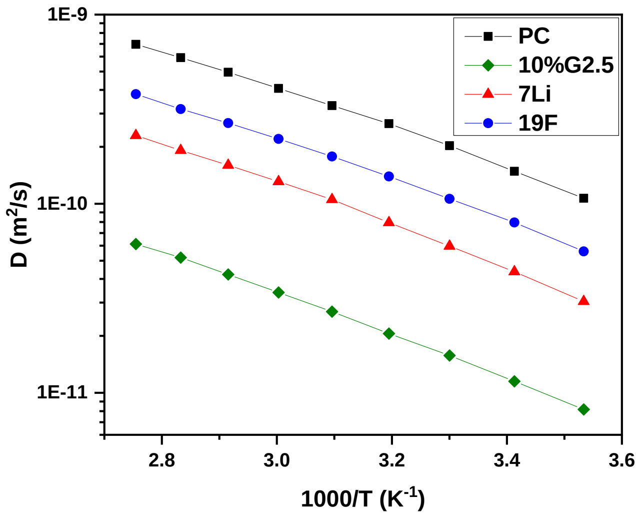

Figure S8. Temperature dependence of NMR self-diffusion coefficients for the LiTFSI_10%G2.5 electrolyte.

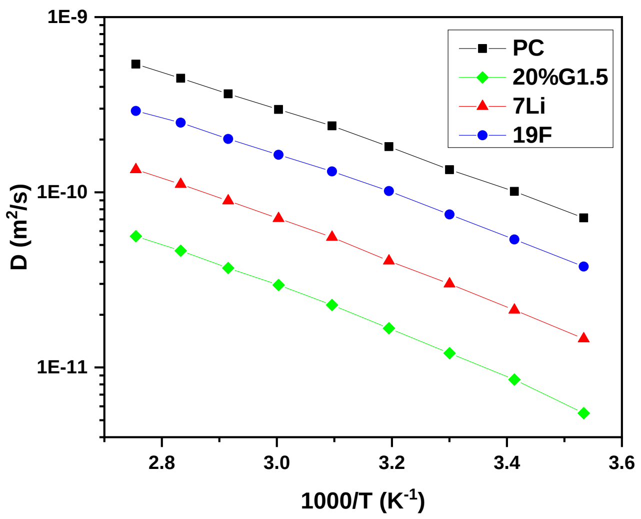

Figure S9. Temperature dependence of NMR self-diffusion coefficients for the LiTFSI_20%G1.5 electrolyte.

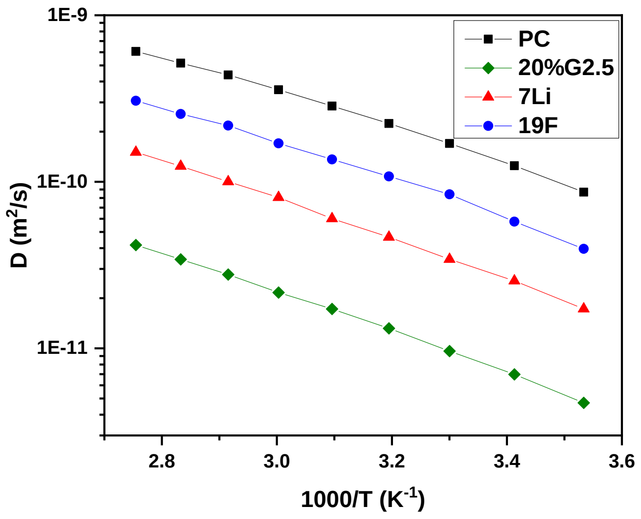

Figure S10. Temperature dependence of NMR self-diffusion coefficients for the LiTFSI_20%G2.5 electrolyte.

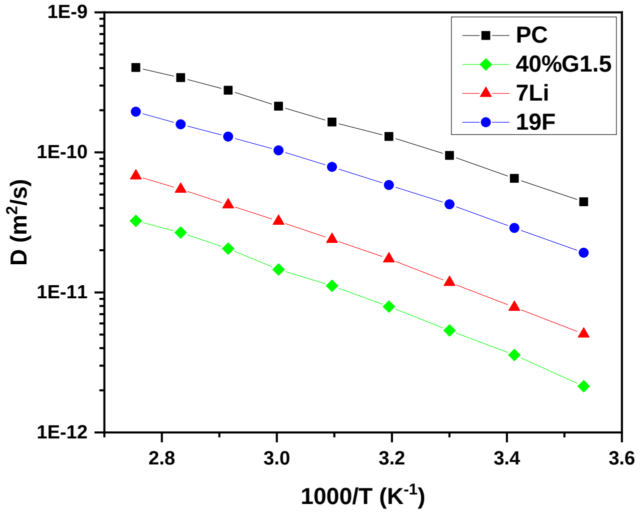

Figure S11. Temperature dependence of NMR self-diffusion coefficients for the LiTFSI_40%G1.5 electrolyte.

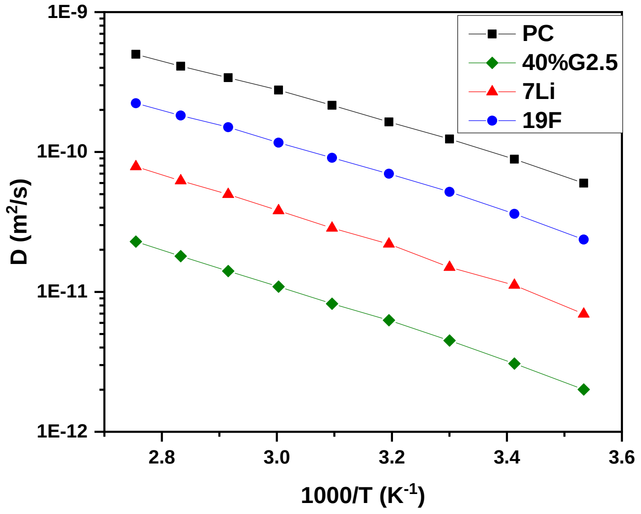

Figure S12. Temperature dependence of NMR self-diffusion coefficients for the LiTFSI_40%G2.5 electrolyte.

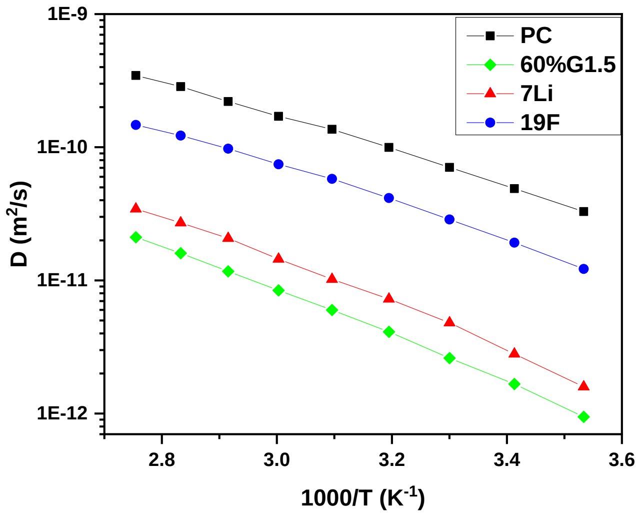

Figure S13. Temperature dependence of NMR self-diffusion coefficients for the LiTFSI_60%G1.5 electrolyte.

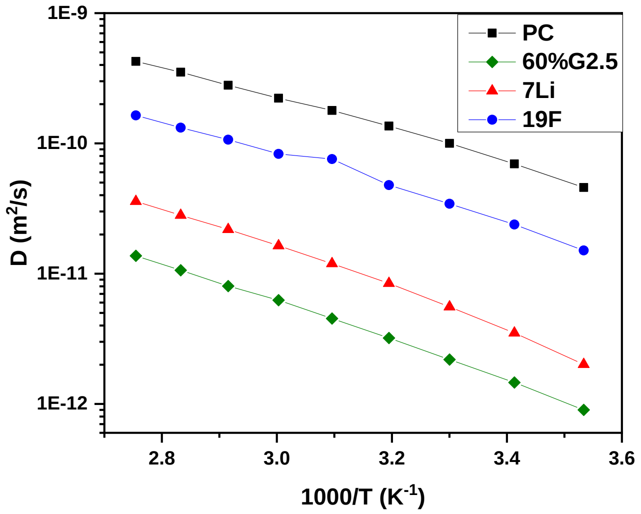

Figure S14. Temperature dependence of NMR self-diffusion coefficients for the LiTFSI_60%G2.5 electrolyte.

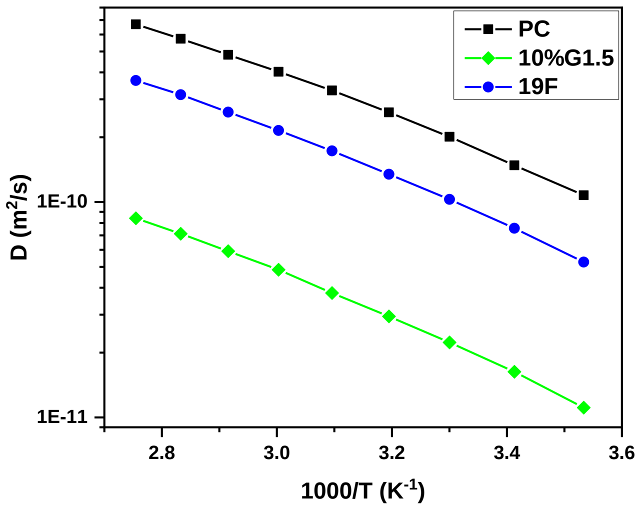

Figure S15. Temperature dependence of NMR self-diffusion coefficients for the NaTFSI_10%G1.5 electrolyte.

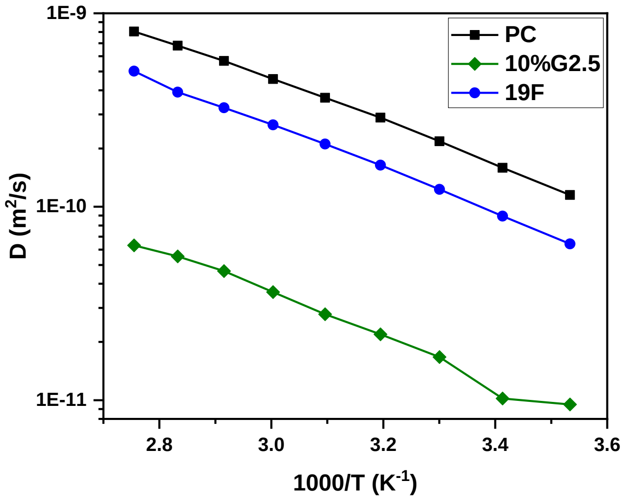

Figure S16. Temperature dependence of NMR self-diffusion coefficients for the NaTFSI_10%G2.5 electrolyte.

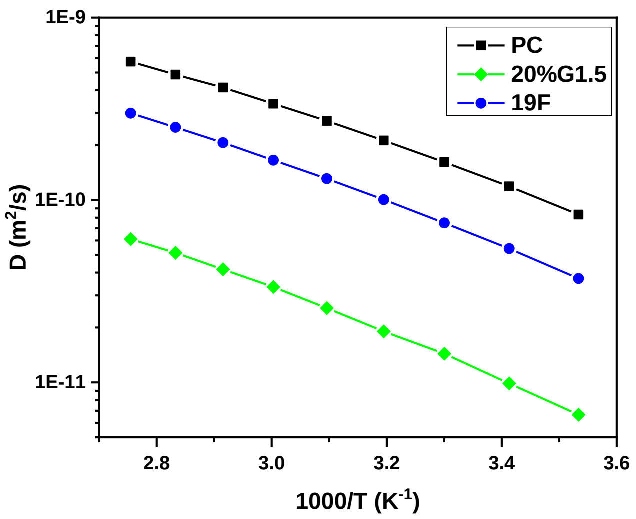

Figure S17. Temperature dependence of NMR self-diffusion coefficients for the NaTFSI_20%G1.5 electrolyte.

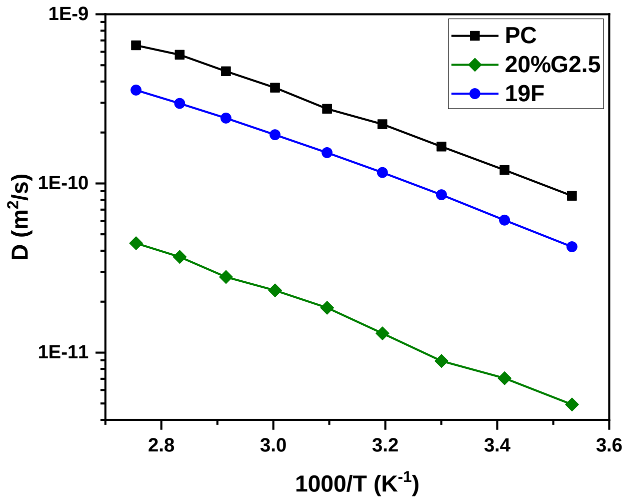

Figure S18. Temperature dependence of NMR self-diffusion coefficients for the NaTFSI_20%G2.5 electrolyte.

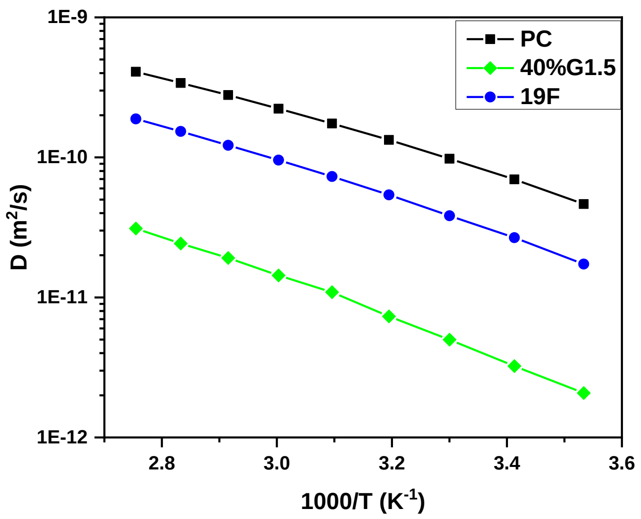

Figure S19. Temperature dependence of NMR self-diffusion coefficients for the NaTFSI_40%G1.5 electrolyte.

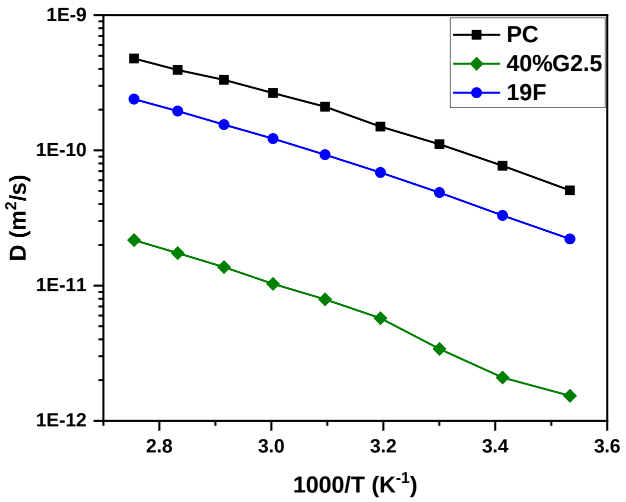

Figure S20. Temperature dependence of NMR self-diffusion coefficients for the NaTFSI_40%G2.5 electrolyte.

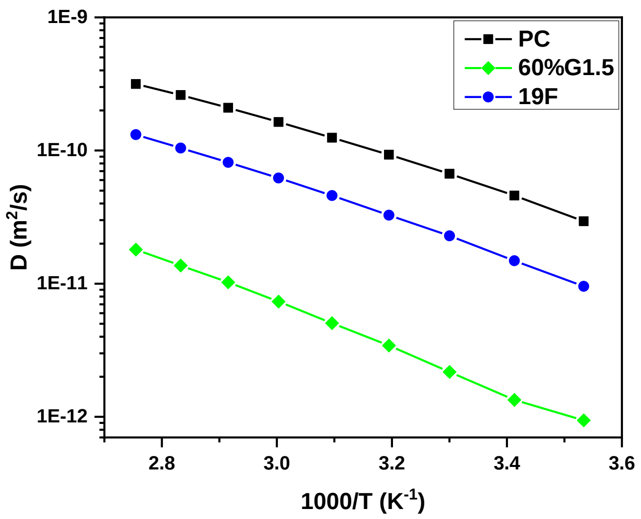

Figure S21. Temperature dependence of NMR self-diffusion coefficients for the NaTFSI_60%G1.5 electrolyte.

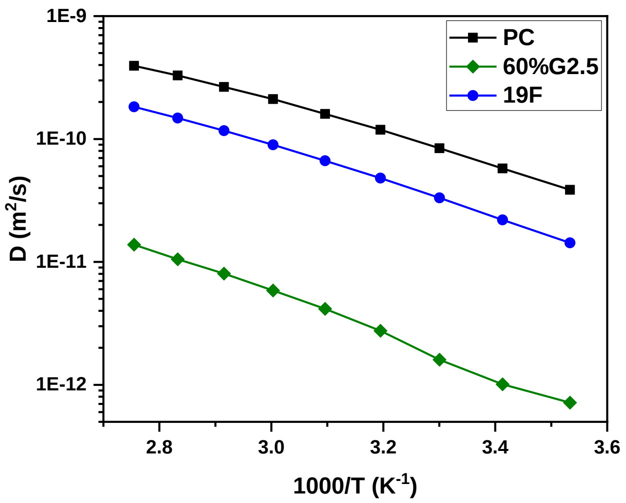

Figure S22. Temperature dependence of NMR self-diffusion coefficients for the NaTFSI_60%G2.5 electrolyte.

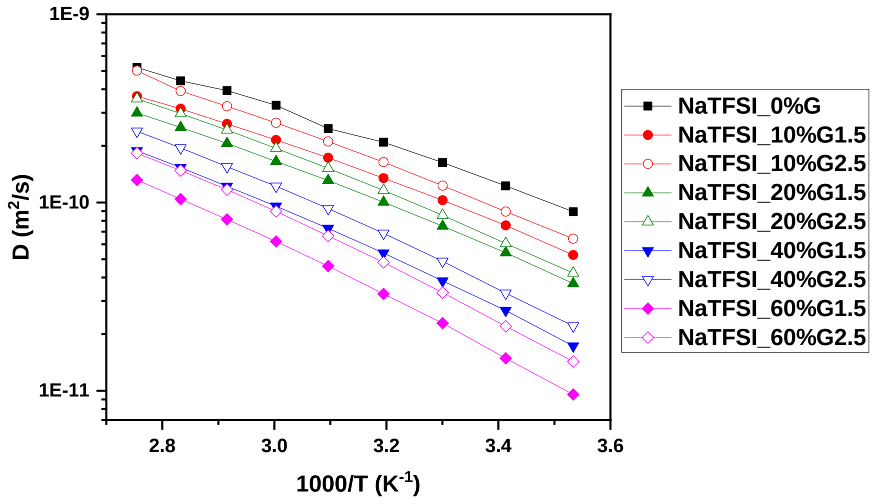

Figure S23. Temperature dependence of NMR self-diffusion coefficients for the NaTFSI based electrolytes for TFSI anion.

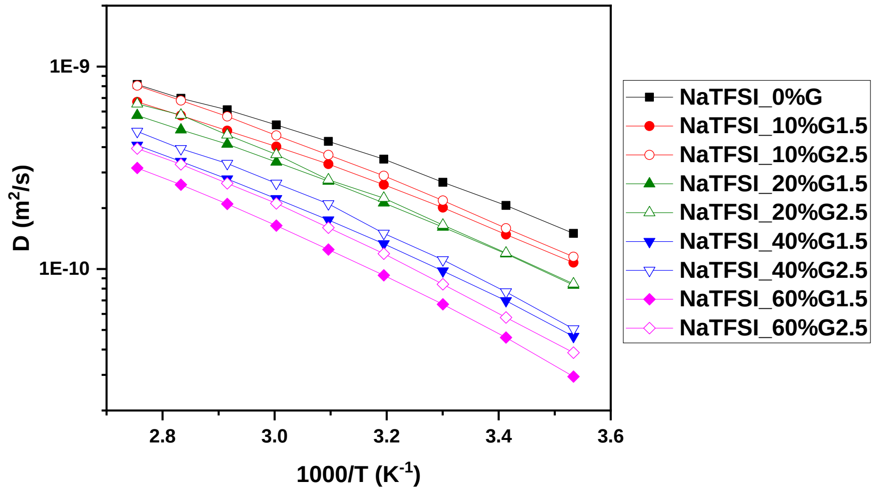

Figure S24. Temperature dependence of NMR self-diffusion coefficients for the NaTFSI based electrolytes for PC.

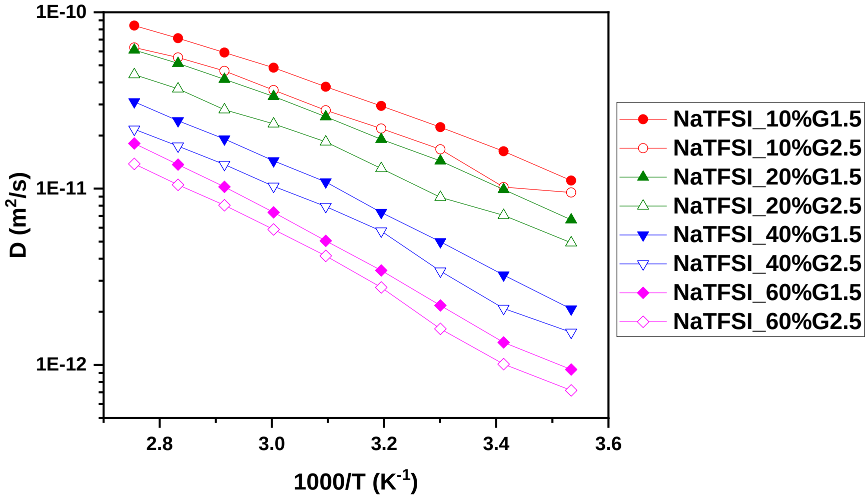

Figure S25. Temperature dependence of NMR self-diffusion coefficients for the NaTFSI based electrolytes for PAMAM.
